# Mental burden among Chinese undergraduate medical students: A prospective longitudinal study before, during, and after the COVID-19 outbreak

**DOI:** 10.3389/fpsyt.2022.982469

**Published:** 2022-10-06

**Authors:** Xiao Liao, Simai Zhang, Yue Wang, Jingwen Jiang, Yuchen Li, Wei Zhang

**Affiliations:** ^1^Mental Health Center, West China Hospital, Sichuan University, Chengdu, China; ^2^West China Biomedical Big Data Center, West China Hospital, Sichuan University, Chengdu, China

**Keywords:** COVID-19, undergraduate medical students, psychological distress, stress reaction, insomnia, longitudinal study

## Abstract

**Background:**

Increasing evidence indicated a clear association between COVID-19 pandemic and mental health. This study aimed to assess the dynamic change of mental burden during and after the COVID-19 outbreak and related predictive factors among Chinese undergraduate medical students.

**Methods:**

This longitudinal survey was conducted among Chinese undergraduate medical students before, during, and after the COVID-19 outbreak. We focused on COVID-19 related mental burdens including psychological distress, stress reaction, and insomnia symptoms, and defined the sum score of the three specific mental burden indexes as the overall mental burden index. The prevalence of specific and overall mental burdens and their changing patterns at two phases of the pandemic (during vs. after the COVID-19 outbreak) were measured. In addition, multinomial logistic regressions were used to assess the associations between the psychosocial status before the pandemic and specific and overall mental burden changing patterns.

**Results:**

Our findings showed that the prevalence of overall mental burden increased (from 27.46 to 37.28%) after the COVID-19 outbreak among the 863 Chinese undergraduate medical students who participated in the surveys at baseline, during, and after the COVID-19 outbreak. Specifically, the prevalence of stress reaction symptoms decreased (from 10.90 to 3.60%), while the rates of psychological distress (from 28.06 to 37.95%) and insomnia symptoms (from 12.54 to 20.71%) increased. Participants, with obsessive-compulsive symptoms, somatic symptoms, internet addiction, childhood adversity, stressful life events, and being neurotic were found to have a higher risk of developing mental burden in at least one survey (during or after the COVID-19 outbreak). Healthy family function and being extravert were found to positively impact mental burden.

**Conclusion:**

Psychological distress, stress reaction and insomnia symptoms have been prevalent among Chinese undergraduate medical students during the COVID-19 outbreak, and the prevalence of overall mental burden increased after the COVID-19 outbreak. Some students, especially those with the risk factors noted above, exhibited persistent or progression symptoms. Continued mental health care was in demand for them even after the COVID-19 outbreak.

## Introduction

In December 2019, the outbreak of the novel coronavirus disease (COVID-19) aroused global attention (1). Until 29th June 2022, 543,352,927 patients have been diagnosed globally and 6,331,059 died from COVID-19 (2). Despite the number of patients infected by COVID-19 is under control in China now (2), we are still fighting the virus. Compelling evidence suggested that the Chinese general population (3), especially healthcare workers (4), showed some mental health symptoms, including psychological distress, depression, anxiety, and insomnia during the COVID-19 outbreak in China. Medical students, whose majors are related to healthcare, have also reported that the risk of psychological problems such as anxiety, depression, and perceived stress increased during the COVID-19 outbreak (5) As a unique group, medical students faced profound challenges during the COVID-19 pandemic. For instance, the high contagiousness of the virus has made it challenging to continue regular lectures, which has affected the medical education process, based on lectures and patient-based education (6). Additionally, the availability of bedside teaching opportunities for medical students was constrained by the limited patient care due to the concentration on COVID-19 patients (7). Other challenge includes a fear that medical students may contract the virus while training and spread it to the community (8). It's also reported that medical students have increased stress during previous pandemics, such as the 2003 SARS outbreak in China, underscoring the need for additional support for them during public health crises (9). Therefore, it is necessary to explore the mental health status among medical students during the COVID-19 pandemic.

Increasing evidence indicated that the outbreak of COVID-19 affected mental health (10). Several two-wave studies have revealed that the rates of mental health symptoms changed (11, 12). Due to the ongoing epidemic, long-term longitudinal research on mental health across time is necessary. Previous studies have indicated that personality traits (13), with a prior history of mental illness (14, 15), internet addiction (16), and a stressful life environment (16, 17) are predictors for a broad range of COVID-19 related mental problems. But little is known about the associations between these different kinds of psychosocial status before the COVID-19 outbreak and mental burden at different stages of the epidemic.

From these observations, there are two questions to be explored. How is the dynamic change of mental burden during and after the COVID-19 outbreak among undergraduate medical students? Which factors can predict the dynamic change? Thus, leveraging a prospective cohort of undergraduate medical students in China, which has collected enriched information on psychosocial status before the COVID-19 outbreak and mental burden during and after the local COVID-19 outbreak, we aimed to assess the mental burden changing patterns during and after the COVID-19 outbreak and identify related predictive factors among undergraduate medical students in advance. It can provide more reference for mental health promoting during the COVID-19 pandemic or other infectious diseases.

## Methods

### Study sample

The study sample was retrieved from the ongoing health professional students' prospective health cohort concerning the psychosocial status of medical students at Sichuan University, China. We invited all undergraduates (2,483 in total) from West China School of Medicine and 2025 undergraduates (81.55%) completed the baseline questionnaires before the unprecedented COVID-19 outbreak (October 2019). During the COVID-19 outbreak in China, we invited all participants for a special assessment of the COVID-19 related mental health and 1,553 undergraduates participated in it from February 7th to 13th, 2020. After excluding 64 undergraduates without information about mental burdens, 1,489 undergraduates (73.53%) were included. During the COVID-19 remission stage in China, 870 undergraduates who volunteered to come back to school after COVID-19 outbreak in China were surveyed from May 6th to June 6th, 2020. After excluding 7 undergraduates without information about mental burdens, 863 undergraduates (42.62%) were included.

**Figure 1:**
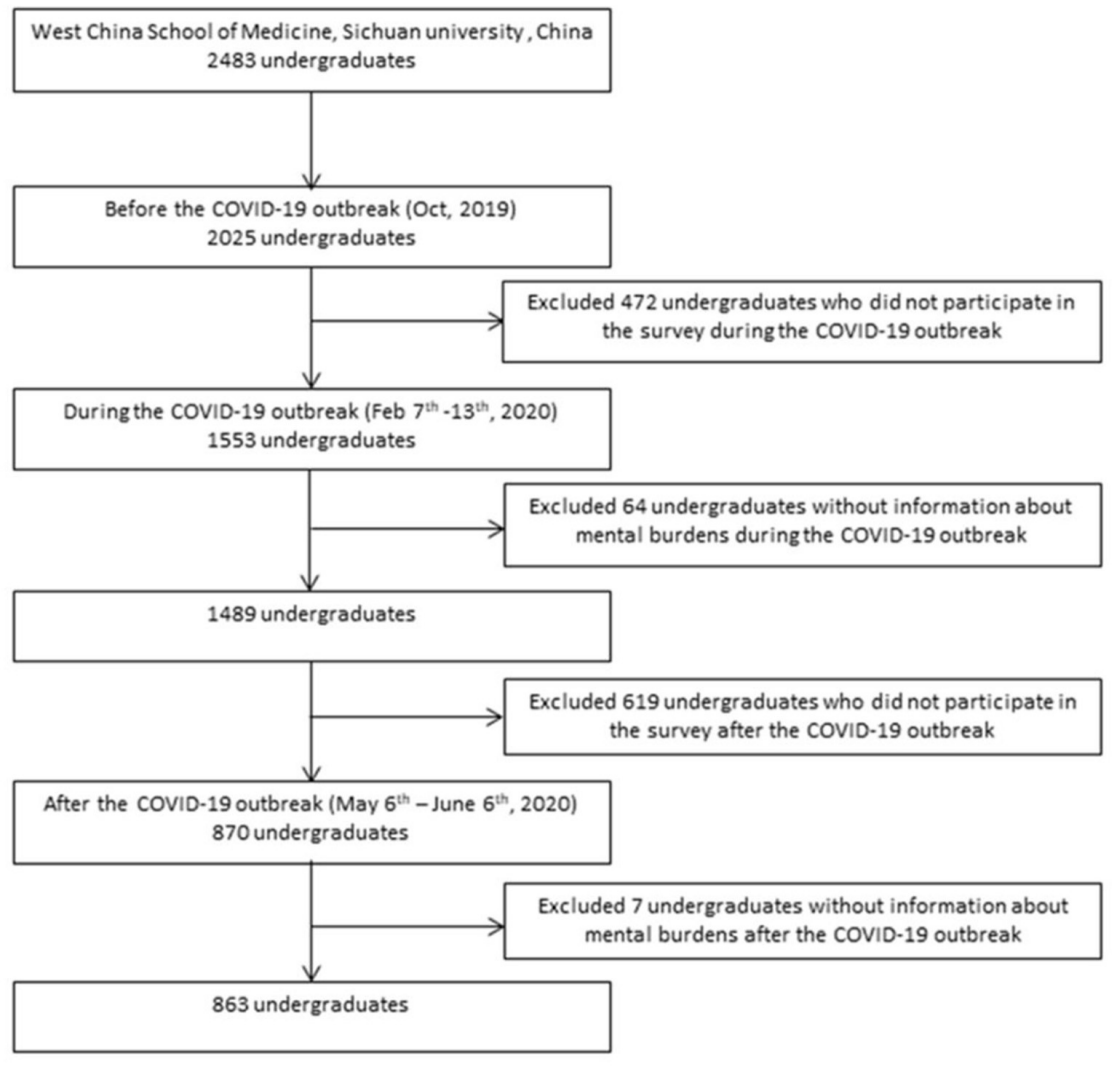
The study flow chart.

The purpose of our assessment was to investigate the psychosocial status of all participating students, and we included data from all undergraduates who have completed the related questionnaires. With a focus on mental burden changing patterns during the whole COVID-19 period, the analysis was restricted to 863 undergraduates who participated in the surveys at baseline, during, and after the COVID-19 outbreak (Figure 1). The questionnaire survey was conducted through a WeChat applet called Psyclub and approved by the Ethics Committee on Biomedical Research, West China Hospital of Sichuan University-2018 Annual Review (No. 535) and 2020 Annual Review (No. 734).

### Measurements

#### Baseline psychosocial status

To observe the baseline psychosocial status, we collected mental health problems (obsessive-compulsive symptoms and somatic symptoms), mental health related behaviors (excessive internet use), environmental status (childhood adversity, stressful life events, and family function), and personality traits (personality and resilience) in October 2019.

All assessments were conducted using web-based, validated questionnaires. Specifically, the obsessive-compulsive subscale of SCL-90 was designed to assess the obsessive-compulsive symptoms. The instrument contained 10 related items and a score ≥2.0 was identified as possibly obsessive-compulsive disorder (18). We used Patient Health Questionnaire-15 (PHQ-15) to assess the severity of somatic symptoms during the past week (19). We identified a possible somatization disorder with a total score ≥5 (20). The 20-item Internet Addiction Test (IAT) was a measure of excessive use of the Internet, and its total scores range from 0-100 and a score ≥31 indicated the mild-above internet addiction (21). Childhood adversity, recent life events, family functioning, and resilience were assessed by the Childhood Trauma Questionnaire-Short Form (CTQ-SF) (22), Adolescent self-rating life events check-list (ALSEC) (23), General functioning subscale of Family Assessment Device (FAD) (24), and the Ego-Resiliency Scale (ER-89) (25), respectively. In addition, the 60-item NEO-Five Factor Inventory (NEO-FFI) was applied to assess the five broad personality domains of neuroticism, extraversion, openness, agreeableness, and conscientiousness (26). Each dimension had 12 items, and its total scores were dichotomized into low and high by the median.

#### Specific mental burden

To explore the COVID-19 related mental burden of participants, we focused on three symptoms, psychological distress, stress reaction, and insomnia, which were the most observed mental health outcomes during the COVID-19 outbreak in healthcare personnel (27). In this study, they were considered as specific mental burdens. Each significant symptom of the below three was defined as 1 point, while non-significance was 0 point, considered as a specific mental burden index.

Psychological distress during and after the COVID-19 outbreak was assessed using the Kessler 6-item Psychological distress Scale (K6). K6 was designed to access the severity of mood disorder symptoms, including major depression and generalized anxiety disorders during the past month (28). The K6 was composed of 6 items, and each item was designed with a 5-point scale (0 = never, 4 = all the time). The total score ranged from 0 to 24 points, and more than 5 points were considered as clinically significant psychological distress. The Cronbach's alpha for the survey during and after the COVID-19 outbreak were 0.91 and 0.93, respectively.

Stress reactions, including COVID-19 related intrusion, avoidance, and hyperarousal symptoms were evaluated by the Impact of Event Scale-Revised (ISE-R). The ISE-R included 22 items, and each item was designed with a 5-point scale (0 = not at all, 4 = extremely). Those with a total score over 24 were considered as significant COVID-19 related stress (29). The Cronbach's alpha for the surveys during and after the COVID-19 outbreak were 0.92 and 0.93, respectively.

Insomnia symptoms were evaluated by the Insomnia Severity Index (ISI). The 7-item ISI measured the self-perceived insomnia symptoms and mental burden degree caused by insomnia during the past two weeks (30). Each item was rated on a five-point Likert scale, and those with a total score over 8 were considered as clinically significant insomnia. The Cronbach's alpha for the surveys during and after the COVID-19 outbreak were 0.86 and 0.86, respectively.

#### Overall mental burden

Besides the above three specific mental burdens, we also developed a rule to evaluate the overall mental burden. The sum score of the above three specific mental burden indexes, ranging from 0–3, was defined as the overall mental burden index, and more than 0 was considered as a significant overall mental burden.

#### Ascertainment of specific mental burden changing patterns

Evidence indicated there were long-term mental health effects of COVID-19 among healthcare workers (31), and mental health problems increased during remission compared with during the onset of the COVID-19 outbreak (17, 32). In order to clarify the dynamics of COVID-19 related mental burden and to find students who are in stable high, aggravated, recovering or stable mild levels, we categorize the entire student population into different groups. We then refer to prior studies (32) to further summarize four changing patterns including persistence, progression, regression, and resilience patterns. To explore the dynamic changes of COVID-19 related mental burden, we defined four specific changing patterns of psychological distress, stress response, and insomnia symptoms by comparing the specific mental burden index during (Feb 2020) and after (May 2020) the COVID-19 outbreak. The four changing patterns include persistence pattern (specific mental burden index was both 1 during and after the COVID-19 outbreak), progression pattern (specific mental burden index was 0 before but 1 after), regression pattern (specific mental burden index was 1 before but 0 after), and resilience pattern (specific mental burden index were both 0).

#### Ascertainment of overall mental burden changing patterns

As for overall mental burden changing patterns, the persistence pattern was assigned when the overall mental burden index during and after the COVID-19 outbreak was equal and both greater than 0. Progression pattern referred to an increase in overall mental burden index (i.e., the sum score measured after the COVID-19 outbreak was higher than that during the COVID-19 outbreak), while regression pattern was defined as the opposite condition (i.e., a decrease in overall mental burden index). Resilience pattern was considered when the overall mental burden index during and after the COVID-19 outbreak were both 0.

#### Covariates

Participants provided demographic information on their age, gender, maternal and paternal educational level, and maternal and paternal occupations. Besides, family environment [from urban (yes or no), having siblings (yes or no) and being a left-behind child (yes or no)], school life [training program (medicine, medical technology or nursing)] and physical condition [body mass index (BMI) (calculated and classified into <18.5, 18.5 to <22.9 (reference), 23.0 to <27.5, and ≥27.5 kg/m^2^)] were surveyed.

#### COVID-19 related infection condition

Be infected with COVID-19 were more likely to have psychological issues (33, 34). To explore the infection condition, we have surveyed whether these students and their relatives had a COVID-19 infection or not in February 2020.

### Statistical analysis

First, we examined the characteristics of all participants and participants in different overall mental burden changing patterns *via* one-way ANOVA (for continuous variables) and Chi-square test (for categorical variables). To explore the loss to follow-up bias, we also compared the differences of characteristics of the loss and the including students. Then, we calculated the prevalence of each specific and overall mental burden during and after the COVID-19 outbreak and in different changing patterns, as well as comparing the differences among three specific mental burdens. Multinomial logistic regressions were used to assess the associations between the baseline psychosocial status and specific and overall mental burden changing patterns, in which the resilience pattern was used as the reference category. Odds Ratios (ORs) with 95% confidence intervals (CIs) were provided. All models were adjusted for demographic information, including age, gender, maternal and paternal educational level, maternal and paternal occupations, family environment, including from urban, having siblings, and being a left-behind child, school life, including training program, and physical condition, including BMI. Additionally, we applied the dose-response analysis to assess the association between the number of identified risk factors and overall mental burden changing patterns. Similarly, the resilience pattern was used as the reference category.

## Results

### Characteristics

The mean age of the total 863 participants whose majors included medicine, medical technology, and nursing was 20.62 years (Table 1). Among them, 61.65 were females, 68.60% were from urban areas, 60.83% were the only child of their family, and 13.79% were left-behind children. Most of their parents owned middle school education level (paternal: 46.81%; maternal: 48.09%) and white-collar occupation (paternal: 38.93%; maternal: 30.71%). Furthermore, there was no difference (*p* > 0.01) among participants in different overall mental burden changing patterns. None reported COVID-19, while 10 (1.15%) had at least one relative infected. When we compared the differences of the characteristics of the loss and the including students, Results indicated that there were significant differences (*p* < 0.01) in age, training program, whether from urban or not, parental educational level and occupation (Supplementary Table S1).

**Table 1:** Characteristics of the 863 undergraduate medical students^a^.

	**Total** ***n* (%)**	**Persistence pattern** ***n* (%)**	**Progression pattern** ***n* (%)**	**Regression pattern** ***n* (%)**	**Resilience pattern *n* (%)**	***F/χ^2^***	**df**	***p***
Characteristics	863 (100)	99 (11.47)	216 (25.02)	133 (15.41)	397 (46.00)			
Age, years (M ± SD)	20.62 ± 1.45	20.53 ± 1.41	20.50 ± 1.41	20.76 ± 1.42	20.60 ± 1.45	1.01	844	0.89
**Gender**								
Female	532 (61.65)	64 (64.65)	135 (62.5)	84 (63.16)	239 (60.2)	0.92	3	0.82
Male	331 (38.35)	35 (35.35)	81 (37.5)	49 (36.84)	158 (39.8)			
**BMI, kg/m** ^2^								
<18.5	148 (17.15)	15 (15.15)	41 (18.98)	19 (14.29)	69 (17.38)	14.51	9	0.11
18.5–22.9	533 (61.76)	64 (64.65)	143 (66.2)	83 (62.41)	232 (58.44)			
23.0–27.5	146 (16.92)	16 (16.16)	23 (10.65)	22 (16.54)	83 (20.91)			
>27.5	36 (4.17)	4 (4.04)	9 (4.17)	9 (6.77)	13 (3.27)			
**Training program**								
Medicine	401 (46.47)	44 (44.44)	93 (43.06)	62 (46.62)	191 (48.11)	2.61	6	0.86
Medical technology	330 (38.24)	38 (38.38)	90 (41.67)	48 (36.09)	150 (37.78)			
Nursing	132 (15.3)	17 (17.17)	33 (15.28)	23 (17.29)	56 (14.11)			
**From urban**								
Yes	592 (68.6)	62 (62.63)	141 (65.28)	103 (77.44)	271 (68.26)	7.52	3	0.06
No	271 (31.4)	37 (37.37)	75 (34.72)	30 (22.56)	126 (31.74)			
**Having siblings**								
Yes	338 (39.17)	47 (47.47)	93 (43.06)	43 (32.33)	151 (38.04)	6.99	3	0.07
No	525 (60.83)	52 (52.53)	123 (56.94)	90 (67.67)	246 (61.96)			
**Being a left-behind child**								
Yes	119 (13.79)	15 (15.15)	35 (16.2)	18 (13.53)	47 (11.84)	2.50	3	0.48
No	744 (86.21)	84 (84.85)	181 (83.8)	115 (86.47)	350 (88.16)			
**Paternal educational level**								
Primary school	102 (11.82)	13 (13.13)	35 (16.2)	16 (12.03)	35 (8.82)	17.45	6	0.01
Middle school	404 (46.81)	39 (39.39)	106 (49.07)	50 (37.59)	200 (50.38)			
College and above	357 (41.37)	47 (47.47)	75 (34.72)	67 (50.38)	162 (40.81)			
**Paternal occupation**								
White collar	336 (38.93)	33 (33.33)	81 (37.5)	59 (44.36)	156 (39.29)	10.31	15	0.80
Blue collar	141 (16.34)	17 (17.17)	33 (15.28)	21 (15.79)	66 (16.62)			
Farmers	130 (15.06)	17 (17.17)	35 (16.2)	18 (13.53)	58 (14.61)			
Self-employed	132 (15.3)	13 (13.13)	32 (14.81)	17 (12.78)	68 (17.13)			
Unemployment	53 (6.14)	10 (10.1)	16 (7.41)	8 (6.02)	17 (4.28)			
Other	71 (8.23)	9 (9.09)	19 (8.8)	10 (7.52)	32 (8.06)			
**Maternal educational level**								
Primary school	167 (19.35)	23 (23.23)	45 (20.83)	21 (15.79)	74 (18.64)	12.51	6	0.05
Middle school	415 (48.09)	42 (42.42)	113 (52.31)	54 (40.6)	197 (49.62)			
College and above	281 (32.56)	34 (34.34)	58 (26.85)	58 (43.61)	126 (31.74)			
**Maternal occupation**								
White collar	265 (30.71)	27 (27.27)	51 (23.61)	57 (42.86)	124 (31.23)	23.30	15	0.08
Blue collar	137 (15.87)	16 (16.16)	39 (18.06)	20 (15.04)	60 (15.11)			
Farmers	147 (17.03)	20 (20.2)	40 (18.52)	13 (9.77)	72 (18.14)			
Self-employed	113 (13.09)	9 (9.09)	28 (12.96)	19 (14.29)	53 (13.35)			
Unemployment	109 (12.63)	13 (13.13)	34 (15.74)	11 (8.27)	51 (12.85)			
Other	92 (10.66)	14 (14.14)	24 (11.11)	13 (9.77)	37 (9.32)			

a 18 (2.09%) individuals did not have the overall mental burden changing pattern indexes, because they missed the measurement of the Kessler 6-item Psychological distress Scale, the Impact of Event Scale-Revised, or the Insomnia Severity Index.

### Overall mental burden changing patterns

There was an increase in the prevalence of significant overall mental burden (from 27.46 to 37.28%) after the outbreak, representing over one-third of participants experienced significant mental burden after the COVID-19 outbreak (Table 2). As for overall mental burden changing patterns, participants in persistence pattern, progression pattern, regression pattern, and resilience pattern were 11.72% (99/863), 25.56% (216/863), 15.74% (133/863), and 46.98% (397/863), respectively.

**Table 2:** Prevalence of the COVID-19 related mental burden during and after the COVID-19 outbreak.

	**Overall Mental burden^a^ Case, *n* (prevalence %)**	**Psychological distress^b^** **Case, *n* (prevalence %)**	**Insomnia^c^ Case, *n* (prevalence %)**	**Stress reaction^d^** **Case, *n* (prevalence %)**	***χ^2^***	**df**	***p***
**During the COVID-19 outbreak**							
Significant	232 (27.46)	241 (28.06)	106 (12.54)	94 (10.90)	108	2	<0.01
Non-significant	613 (72.54)	618 (71.94)	739 (87.46)	768 (89.1)			
**After the COVID-19 outbreak**							
Significant	315 (37.28)	326 (37.95)	175 (20.71)	31 (3.60)	308.99	2	<0.01
Non-significant	530 (62.72)	533 (62.05)	670 (79.29)	831 (96.4)			
**Changing Patterns**							
persistence pattern	99 (11.72)	163 (18.98)	69 (8.17)	17 (1.97)	340.63	6	<0.01
progression pattern	216 (25.56)	163 (18.98)	106 (12.54)	14 (1.62)			
regression pattern	133 (15.74)	78 (9.08)	37 (4.38)	77 (8.93)			
resilience pattern	397 (46.98)	455 (52.97)	633 (74.91)	754 (87.47)			

a 18 (2.09%) individuals did not have the overall mental burden changing pattern indexes, because they missed the measurement of the Kessler 6-item Psychological distress Scale, the Impact of Event Scale-Revised, or the Insomnia Severity Index.

b 4 (0.46%) subjects missed the measurement of the K6 and were not included in the corresponding analysis.

c 18 (2.09%) subjects missed the measurement of the ISI and were not included in the corresponding analysis.

d 1 (0.12%) subject missed the measurement of ISE-R and was not included in the corresponding analysis.

### Specific mental burden changing patterns

As for specific mental burdens, we observed an increase in the prevalence of self-reported significant psychological distress (from 28.06% to 37.95%) and insomnia (from 12.54% to 20.71%) after the COVID-19 outbreak. By contrast, the prevalence of significant stress reaction decreased from 10.90% to 3.60% after the outbreak. Furthermore, there were significant differences (*p* < 0.01) among participants experienced three specific mental burdens during and after the COVID-19 outbreak and in different changing patterns.

For psychological distress, 18.98% of participants were in the persistence pattern, which is equivalent to the proportion in the progression pattern. For insomnia, 8.17% and 12.54% of participants were in persistence and progression patterns, respectively. For stress reaction, 1.97% and 1.62% of participants were in persistence and progression patterns, respectively.

### Predictive factors of mental burden changing patterns

For overall mental burden, with obsessive-compulsive symptoms (OR 3.21, 95% CI 1.99–5.20), somatic symptoms (OR 2.84, 95% CI 1.72–4.67), internet addiction (OR 2.96, 95% CI 1.80–4.86), childhood adversity (OR 3.01, 95% CI 1.83–4.93), stressful life events (OR 3.44, 95% CI 2.10–5.65) and being neurotic (OR 2.92, 95% CI 1.80–4.74) at baseline were positively associated with being in the persistence pattern (Table 3). These predictive factors were also associated with being in the progression and regression pattern, which indicated that the likelihood of developing any mental burden symptoms in at least one wave (“non-resilience pattern”) would increase if the students endorsed these risk factors before the COVID-19 outbreak. On the contrary, healthy family function (OR 0.41, 95% CI 0.25–0.67) and being extravert (OR 0.44, 95% CI 0.27–0.72) were negatively associated with being in the persistence pattern. These predictive factors were also negatively associated with being in the progression and regression pattern, which indicated that the likelihood of developing any mental burden symptoms in at least one wave (“non-resilience pattern”) would decrease if the students endorsed these protective factors before the COVID-19 outbreak. Similar results were observed in psychological distress, insomnia, and stress reaction changing patterns (Supplementary Table S2).

**Table 3:** Predictors associated with the overall mental burden changing patterns of the 863 undergraduate medical students^a^.

	**Persistence pattern OR (95% CI)**	**Progression pattern** **OR (95% CI)**	**Regression pattern OR (95% CI)**	**Resilience pattern** **OR (95% CI)**
Obsessive-compulsive symptoms	3.21 (1.99–5.20)	2.71 (1.88–3.90)	2.89 (1.88–4.47)	Ref.
Somatic symptoms	2.84 (1.72–4.67)	3.37 (2.32–4.91)	2.64 (1.71–4.07)	Ref.
Internet addiction	2.96 (1.80–4.86)	1.78 (1.25–2.54)	1.96 (1.28–2.98)	Ref.
Childhood adversity	3.01 (1.83–4.93)	2.28 (1.58–3.28)	2.36 (1.52–3.66)	Ref.
Stressful life events	3.44 (2.10–5.65)	2.76 (1.93–3.96)	3.19 (2.07–4.89)	Ref.
Family functioning	0.41 (0.25–0.67)	0.43 (0.30–0.62)	0.27 (0.17–0.43)	Ref.
Resilience	0.66 (0.40–1.08)	0.89 (0.62–1.27)	0.83 (0.55–1.27)	Ref.
Neuroticism	2.92 (1.80–4.74)	2.13 (1.49–3.04)	2.56 (1.67–3.90)	Ref.
Extraversion	0.44 (0.27–0.72)	0.68 (0.48–0.97)	0.56 (0.37–0.86)	Ref.
Openness	0.78 (0.48–1.28)	0.79 (0.55–1.13)	0.72 (0.47–1.12)	Ref.
Agreeableness	0.65 (0.39–1.06)	0.81 (0.56–1.16)	0.84 (0.55–1.29)	Ref.
Conscientiousness	0.78 (0.48–1.28)	0.92 (0.64–1.31)	0.98 (0.65–1.49)	Ref.

* *OR*, odds ratio; *CI*, confidence interval; *Ref*, reference.

a 18 (2.09%) individuals did not have the overall mental burden changing pattern indexes, because they missed the measurement of the Kessler 6-item Psychological distress Scale, the Impact of Event Scale-Revised, or the Insomnia Severity Index.

Dose-response relationships were observed between the number of identified psychosocial risk factors (obsessive-compulsive symptoms, somatic symptoms, internet addiction, childhood adversity, stressful life events, and neuroticism) and overall mental burden changing patterns (Table 4). The higher number of identified psychosocial risk factors, the higher risk of being in the overall mental burden persistence pattern, regression pattern, and progression pattern were observed.

**Table 4:** Dose-response analysis between the number of identified baseline psychosocial risk factors and overall mental burden changing patterns.

	**OR (95% CI)^a^**
**Psychosocial risk factors** **=** **1**	
Persistence pattern	1.61 (0.45–5.70)
Regression pattern	1.75 (0.62–4.94)
Progression pattern	2.08 (0.81–5.33)
Resilience pattern	Ref.
**Psychosocial risk factors** **=** **2**	
Persistence pattern	2.98 (0.91–9.77)
Regression pattern	4.08 (1.53–10.90)
Progression pattern	5.12 (2.09–12.54)
Resilience pattern	Ref.
**Psychosocial risk factors** **=** **3**	
Persistence pattern	3.26 (0.98–10.81)
Regression pattern	5.05 (1.89–13.46)
Progression pattern	5.80 (2.39–14.11)
Resilience pattern	Ref.
**Psychosocial risk factors** **=** **4**	
Persistence pattern	4.16 (1.25–13.86)
Regression pattern	5.67 (2.09–15.38)
Progression pattern	8.42 (3.44–20.59)
Resilience pattern	Ref.
**Psychosocial risk factors** **=** **5**	
Persistence pattern	18.46 (5.50–62.00)
Regression pattern	10.66 (3.53–32.16)
Progression pattern	20.76 (7.98–54.00)
Resilience pattern	Ref.
**Psychosocial risk factors** **=** **6**	
Persistence pattern	70.00 (15.96–306.89)
Regression pattern	67.63 (17.31–264.24)
Progression pattern	28.25 (7.51–106.25)
Resilience pattern	Ref.

* *Ref*, reference.

a Estimates were adjusted for age, sex, BMI, training program, family background, being the only child, being a left-behind child, paternal and maternal occupation, and paternal and maternal educational level.

## Discussion

To our best knowledge, this is the first study to explore the changing patterns of COVID-19 related mental burden and its predictive factors among Chinese undergraduate medical students using 3-wave data before, during, and after the COVID-19 outbreak. Our findings showed that the prevalence of overall mental burden increased after the COVID-19 outbreak. Specifically, the prevalence of stress reaction symptoms decreased, while the rates of psychological distress and insomnia symptoms increased from the COVID-19 outbreak to the COVID-19 remission stage in China. The study revealed four changing patterns of mental burden, i.e. persistence pattern, regression pattern, progression pattern, and resilience pattern. Multiple factors, including obsessive-compulsive symptoms, somatic symptoms, internet addiction, childhood adversity, stressful life events, and being neurotic were significant risk factors for overall mental burden persistence pattern, regression pattern, and progression pattern among medical students. Healthy family function and being extravert were significant protective factors of them.

Evidence supported that mental health problems increased during remission compared with during the onset of the COVID-19 outbreak (17, 32). Consistent with previous studies, our study also indicated that the proportion of significant overall mental burden increased after the COVID-19 outbreak. Notably, among the medical students who participated in both surveys, the prevalence of psychological distress (from 28.06% to 37.95%) and insomnia (from 12.54% to 20.71%) increased after the COVID-19 outbreak. This inverse increase phenomenon of psychological distress and insomnia was consistent with previous studies about mental health during the COVID-19 pandemic (17) and longitudinal trajectories of insomnia symptoms among college students (32), respectively. Consistent with related previous studies on college students, our results also indicated that the prevalence of stress reaction symptoms decreased (17). It is expected that the prevalence of acute stress reduced following the epidemic since the IES-R is intended to measure stress reactions to traumatic events (35).

There are some possible explanations about the effect on mental health. For instance, being a medical student during a pandemic may be a stigma at this time due to the contact history with confirmed or suspected COVID-19 patients (36). Previous study also indicated COVID-19-related discrimination is associated with internalized stigma, which in turn predict psychological symptoms over time (37). Moreover, there is direct evidence that COVID-19 affected psychiatry problems and brain function. Previous studies indicated that children infected with COVID-19 were more likely to have psychological issues, such as affective disorders, somatic, internalizing, and externalizing problems by comparing the outcome in the children and pre-schoolers who had COVID-19 and those who did not (33, 34). The hypothalamic-pituitary-adrenal axis, which can interfere with different physiological processes throughout the early stages of development, was affected by the COVID-19 pandemic distress by increasing the creation and release of inflammatory mediators. This imbalance may cause problems with the immunological, endocrine, and nervous systems as well as an increased risk of developing psychiatric illnesses in later life (38). Our results on adolescents can help clear the outcome of COVID-19 on the next generation's mental health.

Previous studies identified that people with a prior history of mental illness are more likely to have greater psychological symptoms during the COVID-19 pandemic (14, 15). Consistent with these results, our study indicated that medical students with obsessive-compulsive symptoms and somatic symptoms were found to have a higher risk of developing mental burden in at least one survey (during or after the COVID-19 outbreak). As for obsessive-compulsive symptoms, those with obsessive-compulsive symptoms may be sensitive to a dangerous situation or a threatening situation (39, 40), resulting in more mental burden especially when exposed to the negative news about COVID-19. As for somatic symptoms, previous studies have reported a significant association between somatic symptoms and psychological outcomes during the COVID-19 pandemic (41, 42). It can be explained that somatic symptoms, such as dyspnea, cough, and headache, are easily confused with COVID-19 symptoms (43), thus increasing individual excessive health attention and even anxiety.

Worth noting that internet addiction was associated with elevated risks of distress and acute stress reaction during the COVID-19 outbreak in our previous finding (16). The current study further indicated that internet addiction was related to an increased risk of mental burden after the COVID-19 outbreak. We assumed that those with internet addiction tended to spend much time on social media, which may serve as a stress source via receiving COVID-19 related negative news from social network updates (44). Besides, to keep the medical education process on track during the lockdown, online lectures were frequently used. Excessive internet users will be less engaged in real life and more concentrated on the internet (45). Therefore, increasing other activities instead of internet use may promote mental health.

The role of environmental status, i. e., childhood adversity, stressful life events, and family function in mental health has already been evidenced in previous COVID-19 studies (16, 17). Our data showed that students with childhood adversity, having stressful life events were significant risk factors for overall mental burden persistence pattern, regression pattern, and progression pattern, which was in line with the previous demonstration that a stressful environment was a risk factor for mental burden after trauma (46, 47). By contrast, it has been indicated that family support after trauma can protect survivors from developing psychological distress (48). Consistent with it, our findings confirmed family function was a protective factor for the mental health of undergraduate medical students after the COVID-19 outbreak. Students with stronger family functioning could receive more family support, promoting successful resiliency in the face of public health catastrophes (49).

Personality traits are predictors for a broad range of COVID-19 related mental problems. Specifically, neuroticism is a risk factor for mental health in the COVID-19 emergency (50), while extraversion is a protective factor (13). Consistent with these, our study further indicated that these two personality traits also influence the mental health status after the COVID-19 outbreak. Importantly, our study indicated undergraduate medical students with more identified psychosocial risk factors have higher risks of persistence and progression patterns of mental health burden. Therefore, we should preferentially offer psychological support and interventions to undergraduate medical students with multiple risk factors.

In sum, our study provides further information on COVID-19 related mental burden particularly among medical students. Mental burdens have been prevalent among them during the COVID-19 outbreak, and even the prevalence of overall mental burden increased during the COVID-19 remission stage in China. We has also identified related predictive factors of COVID-19 mental burden changing patterns, which can provide more reference for mental health preventing of other infectious diseases in the future. Importantly, Medical students were perceived as having professional training and extensive medical knowledge of COVID-19 (51), which raised their awareness of the threats and impending pressure in the early stages of the outbreak. Additionally, medical students were encouraged to participate in the prevention and control of the epidemic (52), which added further difficulties to their already demanding academic schedules. In fact, a recent study of ours found that healthcare students who were more psychologically distressed during the training stage ended up changing their career paths and choosing to work in non-medical industries instead (53). Thus, as a unique group facing so many challenges, continued mental health care was in demand for medical students, especially those with the risk factors noted above, even during the COVID-19 remission stage in China. Both family and medical schools should provide support to improve mental health status and take initiatives directed at reducing excessive internet use among medical undergraduates.

## Strengths and limitations

There are several strengths to this study. First of all, this prospective longitudinal study made a unique contribution to the literature by examining mental burden changing patterns and related predictors among undergraduate medical students using 3-wave data before, during, and after the COVID-19 outbreak. Second, the prevalence of stress reaction declined but psychological distress and insomnia symptoms increased from the early epidemic stage to the remission stage were observed, implying the long-term influence of COVID-19 on mental health. Third, we found different kinds of risk factors for mental burden, including mental health problems, behavior conditions, environmental status, and personality traits. Continued mental health care was in demand for those with the risk factors noted above, even after the COVID-19 outbreak.

Despite the strengths, some limitations should be considered in this study. First of all, we only included 863 students who voluntarily returned to school. Considering the small sample size and the possible selection bias, our study may not provide a thorough mental health profile of undergraduate medical students. Second, there may be some potential impact due to sample loss. More senior, medical technology students were included, perhaps because they had more graduation pressure or clinical work. Third, despite the effect of age, sex, BMI, training program, family background, being the only child, being a left-behind child and socioeconomic status have been adjusted for the analysis, residual confounding such as current location and ever having contact with COVID-19 confirmed patients remain due to the absence of application or collection. Finally, our studied cohort was composed of students at a single medical school. Further studies from more centers with larger samples are expected in the future.

## Conclusion

Our findings indicate that psychological distress, stress reaction, and insomnia symptoms have been prevalent among Chinese undergraduate medical students during the COVID-19 outbreak, and the prevalence of overall mental burden increased after the COVID-19 outbreak. Some students, especially those with the risk factors noted above, exhibited persistent or progression symptoms. Future studies can focus on stress management and psychological interventions for those with multiple risk factors.

## Data availability statement

The original contributions presented in the study are included in the article/Supplementary materials, further inquiries can be directed to the corresponding authors.

## Ethics statement

The studies involving human participants were reviewed and approved by the Ethics Committee on Biomedical Research, West China Hospital of Sichuan University-2018 Annual Review (No. 535) and 2020 Annual Review (No. 734). Written informed consent to participate in this study was provided by the participants' legal guardian/next of kin.

## Author contributions

XL and SZ participated in the study design, data analysis, interpretation of findings, literature search, writing, implementation, and approval of the final manuscript. YL and WZ conceived and designed the study. JJ and YW participated in the study data analysis. All authors have approved the final manuscript.

## Funding

This work was supported by the West China Hospital COVID-19 Epidemic Science and Technology Project (No. HX-2019-nCoV-019 to WZ); and Sichuan University Emergency Grant (No. 2020scunCoVyingji1005 to WZ).

## Conflict of interest

The authors declare that the research was conducted in the absence of any commercial or financial relationships that could be construed as a potential conflict of interest.

## Publisher's note

All claims expressed in this article are solely those of the authors and do not necessarily represent those of their affiliated organizations, or those of the publisher, the editors and the reviewers. Any product that may be evaluated in this article, or claim that may be made by its manufacturer, is not guaranteed or endorsed by the publisher.
