# Supplementary material for: Mental burden among Chinese undergraduate medical students: A prospective longitudinal study before, during, and after the COVID-19 outbreak
Source: Front Psychiatry. 2022 Oct 6;13:982469. doi: 10.3389/fpsyt.2022.982469 (PMC9582608; doi:10.3389/fpsyt.2022.982469)
Supplement: Supplementary file 1 [file Data_Sheet_1.docx]

Supplementary Material

**Table S1. Characteristics of the loss and the including undergraduate medical students.**

|  | Overall | Including ^a^ | Loss ^b^ | t/χ² | *p* |
| --- | --- | --- | --- | --- | --- |
| n | 2025 | 863 | 1162 |  |  |
| Age, years (M±SD) | 20.16±1.55 | 20.62±1.45 | 19.82±1.53 | 11.977 | <0.001 |
| Gender |  |  |  |  |  |
| Female | 1201(59.3) | 532 (61.6) | 669(57.6) | 3.2362 | 0.072 |
| Male | 824(40.7) | 331 (38.3) | 493(42.4) |  |  |
| BMI, kg/m2 |  |  |  |  |  |
| <18.5 | 369(18.2） | 148 (17.1) | 221(19.0) | 3.8162 | 0.282 |
| 18.5-22.9 | 1240(61.2) | 533 (61.8) | 707(60.8) |  |  |
| 23.0-27.5 | 318(5.7) | 146 (16.9) | 172(14.8) |  |  |
| >27.5 | 98(4.8) | 36 (4.2) | 62(5.3) |  |  |
| Training Program |  |  |  |  |  |
| Medicine | 1201(59.4) | 401 (46.47) | 800(68.8) | 104.35 | <0.001 |
| Medical technology | 574(28.3) | 330 (38.24) | 244(21.0) |  |  |
| Nursing | 250(12.3） | 132 (15.3) | 118(10.2) |  |  |
| From urban |  |  |  |  |  |
| Yes | 1442(71.2) | 592 (68.6) | 850(73.1) | 4.7852 | 0.029 |
| No | 583(28.8) | 271 (31.4) | 312(26.9) |  |  |
| Having siblings |  |  |  |  |  |
| Yes | 794(39.2) | 338 (39.2) | 456(39.2) | <0.001 | >0.999 |
| No | 1231(60.8) | 525 (60.8) | 706(60.8) |  |  |
| Being a left-behind child |  |  |  |  |  |
| Yes | 410(26.2) | 119 (13.8) | 291(25.0) | 50.729 | <0.001 |
| No | 1615(79.8) | 744 (86.2) | 871(75.0) |  |  |
| Paternal educational level |  |  |  |  |  |
| Primary school | 233(11.6) | 102 (11.8) | 131(11.4) | 12.198 | 0.002 |
| Middle school | 861(42.7) | 404 (46.8) | 457(39.7) |  |  |
| College and above | 921(45.7) | 357 (41.4) | 564(49.0) |  |  |
| Paternal occupation |  |  |  |  |  |
| White collar | 844(41.7) | 336 (38.9) | 508(43.7) | 15.852 | 0.007 |
| Blue collar | 282(13.9) | 141 (16.3) | 141(12.1) |  |  |
| Farmers | 277(13.7) | 130 (15.1) | 147(12.7) |  |  |
| Self-employed | 351(17.3) | 132 (15.3) | 219(18.8) |  |  |
| Unemployment | 117(5.8) | 53 (6.1) | 64(5.5) |  |  |
| Other | 154(7.6) | 71 (8.2) | 83(7.1) |  |  |
| Maternal educational level |  |  |  |  |  |
| Primary school | 355(17.6) | 167 (19.4) | 188(16.3) | 9.2174 | 0.01 |
| Middle school | 933(46.2) | 415 (48.1) | 518(44.8) |  |  |
| College and above | 730(36.2) | 281 (32.6) | 449(38.9) |  |  |
| Maternal occupation |  |  |  |  |  |
| White collar | 711(35.1) | 265 (30.7) | 446(38.4) | 21.929 | 0.001 |
| Blue collar | 282(13.9) | 137 (15.9) | 145(12.5) |  |  |
| Farmers | 296(14.6) | 147 (17.0) | 149(12.8) |  |  |
| Self-employed | 292(14.4) | 113 (13.1) | 179(15.4) |  |  |
| Unemployment | 248(12.2) | 109 (12.6) | 139(12.0) |  |  |
| Other | 196(9.7) | 92 (10.7) | 104(9.0) |  |  |

a. Including refers to the 863 undergraduates who were included finally.

b. Loss refers to undergraduates who were lost to follow-up.

**Table S2. Predictors associated with specific mental burden changing patterns of the 863 undergraduate medical students.**

|  | Persistence pattern  OR (95% CI) | Progression pattern  OR (95% CI) | Regression pattern  OR (95% CI) | Resilience pattern  OR (95% CI) |
| --- | --- | --- | --- | --- |
| **Psychological distress ^a^** | | | |  |
| Obsessive-compulsive symptoms | 3.96 (2.66 - 5.90) | 2.13 (1.45 - 3.15) | 2.00 (1.18 - 3.39) | Ref. |
| Somatic symptoms | 3.28 (2.16 - 4.97) | 2.74 (1.83 - 4.10) | 1.35 (0.81 - 2.25) | Ref. |
| Internet addiction | 3.63 (2.40 - 5.49) | 1.59 (1.08 - 2.33) | 1.17 (0.70 - 1.96) | Ref. |
| Childhood adversity | 3.22 (2.15 - 4.84) | 1.70 (1.15 - 2.52) | 1.66 (0.98 - 2.82) | Ref. |
| Stressful life events | 3.78 (2.51 - 5.69) | 2.19 (1.49 - 3.21) | 1.82 (1.09 - 3.02) | Ref. |
| Family functioning | 0.27 (0.18 - 0.42) | 0.55 (0.37 - 0.81) | 0.35 (0.20 - 0.59) | Ref. |
| Resilience | 0.59 (0.40 - 0.89) | 0.95 (0.65 - 1.40) | 0.97 (0.58 - 1.61) | Ref. |
| Neuroticism | 3.13 (2.10 - 4.66) | 1.89 (1.29 - 2.77) | 1.86 (1.13 - 3.06) | Ref. |
| Extraversion | 0.47 (0.32 - 0.70) | 0.68 (0.46 – 1.00) | 0.68 (0.41 - 1.14) | Ref. |
| Openness | 0.74 (0.50 - 1.10) | 0.80 (0.54 - 1.19) | 0.68 (0.40 - 1.15) | Ref. |
| Agreeableness | 0.72 (0.48 - 1.07) | 0.81 (0.54 - 1.20) | 0.66 (0.39 - 1.12) | Ref. |
| Conscientiousness | 0.78 (0.53 - 1.16) | 0.81 (0.55 - 1.20) | 1.06 (0.64 - 1.75) | Ref. |
| **Insomnia ^b^** | | | |  |
| Obsessive-compulsive symptoms | 3.42 (1.97 - 5.94) | 2.28 (1.47 - 3.53) | 1.47 (0.70 - 3.08) | Ref. |
| Somatic symptoms | 4.76 (2.51 - 9.02) | 2.41 (1.51 - 3.85) | 2.91 (1.31 - 6.48) | Ref. |
| Internet addiction | 2.73 (1.54 - 4.82) | 1.75 (1.12 - 2.73) | 1.46 (0.70 - 3.04) | Ref. |
| Childhood adversity | 4.02 (2.23 - 7.25) | 1.85 (1.19 - 2.90) | 2.78 (1.32 - 5.87) | Ref. |
| Stressful life events | 4.49 (2.44 - 8.24) | 2.06 (1.32 - 3.21) | 2.36 (1.14 - 4.87) | Ref. |
| Family functioning | 0.30 (0.17 - 0.56) | 0.45 (0.28 - 0.72) | 0.57 (0.27 - 1.20) | Ref. |
| Resilience | 0.83 (0.48 - 1.44) | 0.80 (0.51 - 1.25) | 0.57 (0.27 - 1.20) | Ref. |
| Neuroticism | 2.55 (1.45 - 4.48) | 1.37 (0.88 - 2.12) | 1.51 (0.74 - 3.11) | Ref. |
| Extraversion | 0.46 (0.26 - 0.82) | 0.85 (0.55 - 1.32) | 0.48 (0.22 - 1.04) | Ref. |
| Openness | 0.68 (0.39 - 1.19) | 0.67 (0.42 - 1.07) | 0.50 (0.22 - 1.15) | Ref. |
| Agreeableness | 0.72 (0.41 - 1.28) | 1.09 (0.70 - 1.70) | 1.77 (0.86 - 3.68) | Ref. |
| Conscientiousness | 0.95 (0.55 - 1.64) | 1.00 (0.64 - 1.56) | 0.75 (0.35 - 1.61) | Ref. |
| **Stress reaction ^c^** | | | |  |
| Obsessive-compulsive symptoms | 4.79 (1.42 - 16.15) | 18.9 (3.27 - 109.18) | 2.02 (1.21 - 3.38) | Ref. |
| Somatic symptoms | 1.88 (0.56 - 6.27) | 6.11 (1.43 - 26.22) | 2.02 (1.17 - 3.49) | Ref. |
| Internet addiction | 2.31 (0.71 - 7.50) | 6.24 (1.52 - 25.71) | 1.90 (1.13 - 3.20) | Ref. |
| Childhood adversity | 5.37 (1.54 - 18.73) | 4.19 (1.06 - 16.62) | 1.79 (1.06 - 3.03) | Ref. |
| Stressful life events | 3.09 (0.86 - 11.06) | 19.00 (2.10- 172.21) | 2.80 (1.63 - 4.82) | Ref. |
| Family functioning | 0.26 (0.06 - 1.07) | 0.55 (0.16 - 1.89) | 0.45 (0.26 - 0.79) | Ref. |
| Resilience | 0.82 (0.25 - 2.63) | 1.07 (0.32 - 3.57) | 0.69 (0.41 - 1.18) | Ref. |
| Neuroticism | 1.84 (0.60 - 5.62) | 1.17 (0.35 - 3.92) | 2.23 (1.31 - 3.79) | Ref. |
| Extraversion | 0.39 (0.11 - 1.35) | 1.02 (0.31 - 3.38) | 0.83 (0.49 - 1.39) | Ref. |
| Openness | 0.53 (0.15 - 1.82) | 1.27 (0.37 - 4.30) | 0.66 (0.39 - 1.13) | Ref. |
| Agreeableness | 0.14 (0.03 - 0.72) | 1.55 (0.48 - 4.95) | 1.07 (0.64 - 1.81) | Ref. |
| Conscientiousness | 0.71 (0.2 - 2.47) | 1.02 (0.30 - 3.39) | 1.25 (0.75 - 2.07) | Ref. |

^*^Ref, reference.

a. 4 (0.46%) subjects missed the measurement of the K6 and were not included in the corresponding analysis.

b. 18 (2.09%) subjects missed the measurement of the ISI and were not included in the corresponding analysis.

c. 1 (0.12%) subject missed the measurement of ISE-R and was not included in the corresponding analysis.
